# *In vitro* Determination of Extracellular Proteins from *Xylella fastidiosa*

**DOI:** 10.3389/fmicb.2016.02090

**Published:** 2016-12-26

**Authors:** Juliano S. Mendes, André S. Santiago, Marcelo A. S. Toledo, Maria A. C. Horta, Alessandra A. de Souza, Ljubica Tasic, Anete P. de Souza

**Affiliations:** ^1^Centro de Biologia Molecular e Engenharia Genética, Universidade Estadual de CampinasCampinas, Brazil; ^2^Instituto Agronômico, Centro de Citricultura Sylvio MoreiraCordeirópolis, Brazil; ^3^Departamento de Química Orgânica, Instituto de Química, Universidade Estadual de CampinasCampinas, Brazil; ^4^Departamento de Biologia Vegetal, Instituto de Biologia, Universidade Estadual de CampinasCampinas, Brazil

**Keywords:** *Xylella fastidiosa*, extracellular proteins, biofilm, calcium, LC-MS/MS, PW medium

## Abstract

The phytopathogen *Xylella fastidiosa* causes economic losses in important agricultural crops. Xylem vessel occlusion caused by biofilm formation is the major mechanism underlying the pathogenicity of distinct strains of *X. fastidiosa*. Here, we provide a detailed *in vitro* characterization of the extracellular proteins of *X. fastidiosa*. Based on the results, we performed a comparison with a strain J1a12, which cannot induce citrus variegated chlorosis symptoms when inoculated into citrus plants. We then extend this approach to analyze the extracellular proteins of *X. fastidiosa* in media supplemented with calcium. We verified increases in extracellular proteins concomitant with the days of growth and, consequently, biofilm development (3–30 days). Outer membrane vesicles carrying toxins were identified beginning at 10 days of growth in the 9a5c strain. In addition, a decrease in extracellular proteins in media supplemented with calcium was observed in both strains. Using mass spectrometry, 71 different proteins were identified during 30 days of *X. fastidiosa* biofilm development, including proteases, quorum-sensing proteins, biofilm formation proteins, hypothetical proteins, phage-related proteins, chaperones, toxins, antitoxins, and extracellular vesicle membrane components.

## Introduction

*Xylella fastidiosa* (*X. fastidiosa*) is a Gram-negative xylem-inhabiting bacterium and the causal agent of citrus variegated chlorosis (CVC) in citrus plants in Brazil and other crop diseases worldwide (Lambais et al., 2000; Hopkins and Purcell, 2002; Saponari et al., 2013). *X. fastidiosa* is often transmitted by insects from the family Cicadellidae (sharpshooter leafhoppers) and the family Aphrophoridae (meadow spittlebug) in olive orchards (Cornara et al., 2016). The main pathogenic mechanism of CVC is vascular occlusion caused by bacterial movement and systemic biofilm formation, which cause nutritional deficiencies and hydric stress and subsequently affect plant growth and development (Purcell, 1982; Hopkins and Purcell, 2002; Silva-Stenico et al., 2009).

Studies have shown that bacterial biofilm formation involves different developmental phases, starting from the bacterial planktonic stage (Sauer et al., 2002; Stoodley et al., 2002; Sauer, 2003). *X. fastidiosa*, subsp. pauca strain 9a5c, which is the causal agent of CVC, exhibits distinct stages of growth at the abiotic surface, such as the reversible attachment of cells to the surface through non-specific electrostatic interactions (Martínez and Vadyvaloo, 2014; Janissen et al., 2015), the irreversible attachment of cells caused by an increase in extracellular polymeric substances (EPSs), the beginning of biofilm maturation, maturation of the biofilm architecture between 15 and 20 days of growth, and cell dispersion to the planktonic phase between days 25 and 30 of growth (de Souza et al., 2004; Caserta et al., 2010).

Bacterial biofilms are formed by a complex and intricate architecture that provides protection against a wide range of agents that are antagonistic to the bacteria, thereby facilitating pathogen resistance and dispersal within the host (Costerton et al., 1999; Davey and O’Toole, 2000; Stoodley et al., 2002; Visick, 2009). Many virulence factors required for biofilm growth are under the control of diffusible signal factors (DSFs) in an intercellular communication system known as quorum sensing (QS) (Marques et al., 2002; Chatterjee et al., 2008; LaSarre and Federle, 2013; Kumar et al., 2015).

Among the most important pathogenicity factors in *X. fastidiosa* that promote xylem vessel occlusion via biofilm formation are fimbrial and non-fimbrial adhesins as well as toxins and extracellular enzymes that complement virulence and are dispersed throughout the infected plant (Simpson et al., 2000; Chatterjee et al., 2008). Adhesins are important components that contribute to surface attachment, cell-to-cell aggregation and twitching motility in *X. fastidiosa* (Feil et al., 2007; Cruz et al., 2014) as well as to biofilm formation for other bacteria, such as *Pseudomonas aeruginosa* (O’Toole and Kolter, 1998). Fimbrial adhesins are composed of heteropolymers of several subunits that form appendages called pili or attachment pili, whereas non-fimbrial adhesins are composed of a single protein that is regulated according to the cell density (Gerlach and Hensel, 2007; Caserta et al., 2010; Killiny and Almeida, 2014). There are diverse categories of toxins (T), and many are co-expressed with a specific antitoxin (A) to form a toxin-antitoxin system (TA) that functions in stress responses, programmed cell death and cell growth and death regulation (Hayes, 2003; Gerdes et al., 2005; Yamaguchi and Inouye, 2011; Wen et al., 2014). Studies of *X. fastidiosa* have shown that certain toxins, such as RelE and MqsR, and antitoxins are involved in the regulation of population levels, cell persistence and fitness, biofilm formation, and pathogenicity (Muranaka et al., 2012; Lee et al., 2014; Merfa et al., 2016).

Once inside the xylem vessel, *X. fastidiosa* disperses through the channels that connect the vessels, which are called bordered pits (Chatterjee et al., 2008). These channels allow the passage of xylem sap and protect the plant against embolism; however, the pit membrane blocks the passage of larger structures, such as bacteria, and thus functions as a microfilter (Chatterjee et al., 2008; Jansen et al., 2009; Pérez-Donoso et al., 2010). These pit membranes are composed of cellulose, hemicellulose, pectin, lignin, and proteins (Fry et al., 1994). Previous studies have associated the participation of secreted proteins, such as serine and metallo-protease, endo-1,4-β-glucanase and polygalacturonase with pit membrane degradation, which facilitates the crossing of these membranes by *X. fastidiosa* to reach adjacent vessels (Fedatto et al., 2006; Pérez-Donoso et al., 2010). In addition to secretion systems, Gram-negative bacteria exhibit outer membrane vesicles (OMVs) that serve as secretory vehicles for proteins and lipids (Kuehn and Kesty, 2005; Tseng et al., 2009; Costa et al., 2015). A study on the Temecula strain of *X. fastidiosa* identified an abundant lipase/esterase (LesA) secreted by OMVs that is directly responsible for Pierce’s disease symptoms in grape leaves (Nascimento et al., 2016), and the release of OMVs is inversely proportional to the cell attachment frequency in xylem vessels (Ionescu et al., 2014).

The main objective of this study was to provide a quantitative and descriptive analysis of the extracellular proteins of the strain 9a5c, the causal agent of CVC, and strain J1a12, which cannot form a thick biofilm and induce CVC symptoms when inoculated into citrus plants (Koide et al., 2004). Thus, we hope to present new insights to understand biofilm formation by *X. fastidiosa* by observing the content of extracellular proteins under *in vitro* conditions. We identified *X. fastidiosa* extracellular proteins that are expressed during growth from plankton to a mature biofilm. To achieve this goal, we analyzed extracellular proteins from periwinkle wilt (PW) medium collected on five different days of growth (3, 5, 10, 20, and 30 days), which is equivalent to the cycle of development of *X. fastidiosa in vitro* (planktonic to biofilm phase) as described by Caserta et al. (2010) and de Souza et al. (2004). We also analyzed the extracellular proteins of *X. fastidiosa* in response to a supplemental concentration of calcium (II) chloride.

## Materials and Methods

### Strains, Growth Conditions, and Extraction of the Extracellular Fraction

**FIGURE 1:**
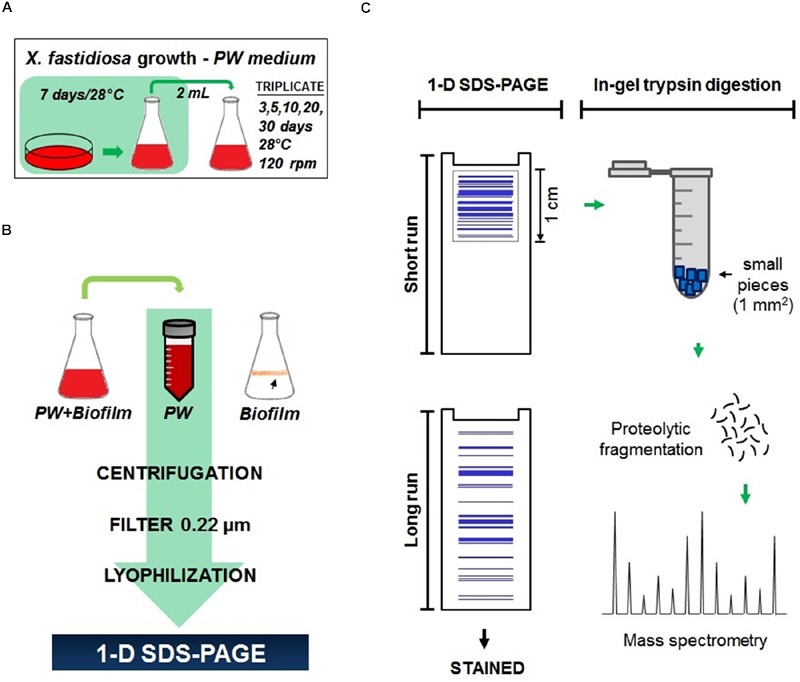
**Schematic representation of the method used to obtain *Xylella fastidiosa* secreted proteins. (A)** Cell growth and propagation in periwinkle wilt (PW) medium. **(B)** Collection, concentration, and purification of PW medium containing extracellular proteins. **(C)** 1D SDS-PAGE analysis and treatment for the mass spectrometry analysis.

The *X. fastidiosa* subsp. pauca (Schaad et al., 2004) strains 9a5c and J1a12, which were isolated from sweet orange trees [*Citrus sinensis* (L.) Osb.], were obtained from the Centro APTA Citros Sylvio Moreira/IAC, Cordeirópolis, Brazil. The bacteria were grown in solid PW medium (Davis et al., 1981) for 7 days at 28°C and then transferred to PW broth for 7 days at 28°C, and they were subjected to rotary agitation at 120 rpm until an *A*_600_ of 0.7 was achieved (**Figure 1A**). Bovine serum albumin (BSA) is frequently added to PW medium; however, in this study, it was removed to avoid interference during the mass spectrometry analyses. BSA is not an essential component for cell growth. A total volume of 2 mL of each suspension with normalized absorbance was inoculated into 125 mL flasks containing 50 mL of PW broth medium. The cultures were incubated at 28°C with rotary agitation at 120 rpm for 3, 5, 10, 20, and 30 days (**Figure 1A**), which was followed by an experimental analysis according to the methods of Caserta et al. (2010) to evaluate extracellular proteins at different stages of the bacterial life cycle from the planktonic to biofilm growth stages. Extracellular proteins were also assessed over 10 days of cultivation on PW medium supplemented with 2.5 mmol dm^-3^ calcium (II) chloride. The corresponding control consisted of *X. fastidiosa* grown without calcium supplementation over the same 10-day period. After this step, the PW medium was centrifuged (10 min; 4°C; 9,000 rpm) and then filtered through a 0.22 μm membrane to remove any suspended cells. The medium containing only extracellular proteins was then lyophilized and stored at -20°C until analysis (**Figure 1B**). All of the growth stages and conditions were analyzed in triplicate.

### SDS-PAGE and In-Gel Trypsin Digestion

The lyophilized extracellular extracts (50 mL) were reconstituted in 5.0 mL of ultra-pure water. An aliquot containing 5 μg of the extract was loaded in triplicate onto a 12% SDS-polyacrylamide gel (Laemmli, 1970) and then run at 120 V. For profile detection, the samples were loaded, separated completely, and visualized by staining with colloidal Coomassie blue (8% ammonium sulfate, 0.8% phosphoric acid, 0.08% Coomassie blue G-250, and 20% methanol). Mass spectrometry was performed after a short run of only 1 cm, and the entire area (sample) was excised (**Figure 1C**). Each sample was sliced into smaller pieces of approximately 1 mm^2^, washed three times with a solution containing 25 mmol dm^-3^ ammonium bicarbonate and 65% acetonitrile at pH 8.0, and destained. The sliced gel pieces were incubated with a reducing agent (10 mmol dm^-3^ dithiothreitol and 25 mmol dm^-3^ ammonium bicarbonate) at 56°C for 1 h, alkylated with a solution containing 10 mmol dm^-3^ iodoacetamide and 25 mmol dm^-3^ ammonium bicarbonate for 45 min at room temperature in the dark, and dehydrated with 100% acetonitrile. The samples were digested with 20 ng of trypsin (Trypsin Gold, Promega, Madison, WI, USA) for 16 h at 37°C. The peptides were extracted with 50% acetonitrile, dried in a speed vacuum (Concentrator 5301, Eppendorf, Hamburg, Germany), and stored at -4°C. All of the analyses were performed in triplicate.

### Cloning, Expression, and Purification of Antitoxin

The XF2491 ORF from LBI-ID encoding the antitoxin HTH-type transcriptional regulator (402 bp; NCBI no. AAF85289.1) was amplified from the genomic DNA of *X. fastidiosa* subsp. pauca strain 9a5c using specific primers (*NdeI*-F: 5′-CAAGGACATATGACCATGAGATGTCC-3′; *XhoI*-R:5′-ACGGCTCGAGACTCTTCACTTCG-3′). The amplification product was cloned into pET29a and used to transform competent C43 (DE3) cells. The cells were grown at 37°C with shaking at 250 rpm until an *A*_600_ of 0.6 was achieved. The antitoxin was expressed with 5.6 mmol L^-1^ inductor lactose for 12 h at 25°C with shaking at 250 rpm. The cells were disrupted by sonication cycles in buffer containing 25 mmol dm^-3^ sodium phosphate at pH 7.8, 150 mmol dm^-3^ NaCl, and 20 mmol dm^-3^ β-mercaptoethanol. The proteins were purified from the supernatant using a Ni-NTA column, and the protein was eluted using imidazole gradients. The purified protein was used to synthesize specific anti-rabbit IgG polyclonal antibodies against the antitoxin HTH-type transcriptional regulator, which was synthesized by Rhea Biotech, Brazil.

### Isolation of OMVs: Transmission Electron Microscopy, Protein Extraction, and Western Blot Analysis

Vesicle separation was performed according to the method of Voegel et al. (2010). Briefly, PW media containing the bacteria were centrifuged and filtered through a 0.22 μm membrane to remove any cells in suspension. *X. fastidiosa* is a rod-shaped bacterium with a radius of 0.25–0.35 μm and a length of 0.9–3.5 μm (Wells et al., 1987). The filtrate was centrifuged at 100,000 × *g* for 4 h at 6°C (L8-80M Ultracentrifuge, Beckman). The supernatant was removed, and the pellet was washed twice with ultra-pure water and centrifuged for 2 h at 100,000 × *g*. For the transmission electron microscopy analysis, the pellet containing OMVs was suspended in 40 mL of ultra-pure water. Formvar-carbon coated 200 mesh copper grids (Ted Pella^®^, Redding, CA, USA) were used to visualize the OMVs based on the method of Nevot et al. (2006). First, the samples were adsorbed onto grids by immersion in 20 mL of the sample for 5 min. The grids were then washed by floating in 20 mL deionized water for 2 min. Finally, the samples were negatively stained by floating the grids on a drop of 2% (w.v^-1^) uranyl acetate for 5 min. After drying for 24 h, the OMVs were visualized using a LEO 906 transmission electron microscope at 60 kV and 167,000 magnification.

Protein extraction from the vesicle lumen and the Western blot analysis were performed using pellets containing OMVs that were treated with 1.5 ng cm^-3^ lysozyme for 10 min and homogenized in buffer containing 0.1% β-mercaptoethanol, 0.0005% bromophenol blue, 10% glycerol, 2% SDS, and 63 mmol dm^-3^ Tris-HCl at pH 6.8. Proteins were extracted using three cycles of sonication for 5 s (Cole Parmer ultrasonic homogenizer 4710 series). The samples were centrifuged, and the supernatants containing OMV proteins were collected. The protein contents were quantified using a Pierce BCA Protein Assay Kit (Rockford, IL, USA). A total of 3.0 μg of proteins were separated by 12.5% SDS-PAGE. The acrylamide gel was then transferred to a nitrocellulose membrane using a Semi-Dry Transfer Cell (Bio-Rad, Hercules, CA, USA). The membrane was blocked in 1% casein solution and incubated first with a specific antibody against antitoxin (dilution 1:1,000) and then with a secondary antibody, anti-rabbit IgG conjugated to the alkaline phosphatase enzyme (dilution 1:8,000) (Rhea Biotech, Brazil). BCIP/NBT Color Development Substrate (Promega) was used to detect alkaline phosphatase activity and visualize the bands.

### Mass Spectrometry

Mass spectrometry analyses were performed on an ESI-Q-TOF Micro Mass Spectrometer coupled to an ultra-performance liquid chromatography system (nanoACQUITY UPLC, Waters). The peptides were separated on two C18 reversed phase columns connected in series (180 μm × 20 mm and 5 μm in particle size; and 100 μm × 100 mm and 1.7 μm in particle size) with an acetonitrile gradient (1–85%) at a flow rate of 0.6 μL min^-1^ for 50 min. The peptides were ionized under 3,000 V and fragmented at 20 V up to 95 V according to the *m/z*, the size of the peptides, and the charged state from 2+ up to 4+. Database searches for *X. fastidiosa* (47,281 sequences; 13,752,578 residues) were performed using Mascot (v 2.3.02). The search parameters were as follows: enzyme: trypsin; maximum missed cleavages: 1; fixed modifications: carbamidomethyl (C); variable modification: methionine oxidation; peptide mass tolerance: 0.1 Da; fragment mass tolerance: 0.3 Da; mass values: monoisotopic; significance threshold: 0.05; and MudPIT protein scoring: 1. All of the mass spectrometry procedures were performed at the Brazilian Synchrotron Light Laboratory (LNLS), Campinas, Brazil. A list of all of the identified proteins containing Minimal Information About a Proteomics Experiment (MIAPE) is provided in the Supplementary Data Sheets 1–3.

### Data Bank Analyses, Sequence Predictions, and Graphical Output

The LBI-ID data bank (annotation) was searched against the *X. fastidiosa* Genome Project^1^. The LBI-ID nomenclature will be used in the text description. The LNCC data bank (re-annotation) was searched against the *X. fastidiosa* comparative database of the *Laboratório Nacional de Computação Científica* (*LNCC*^2^). UniProt accession numbers were retrieved from http://www.uniprot.org/, and secreted proteins were predicted using the following servers: SignalP 4.1 server, http://www.cbs.dtu.dk/services/SignalP; Twin-arginine signal peptide cleavage sites in bacteria (TatP 1.0 server), http://www.cbs.dtu.dk/services/TatP; SecretomeP 2.0 server, http://www.cbs.dtu.dk/services/SecretomeP. Grouping according to gene ontology terms was performed using the Web Gene Ontology Annotation Plot (WEGO), http://wego.genomics.org.cn/. The intersections of Venn diagrams were calculated using a specific tool at the Bioinformatics and Evolutionary Genomics server, http://bioinformatics.psb.ugent.be/webtools/Venn/.

## Results

### Extracellular Proteins of *X. fastidiosa*

**FIGURE 2:**
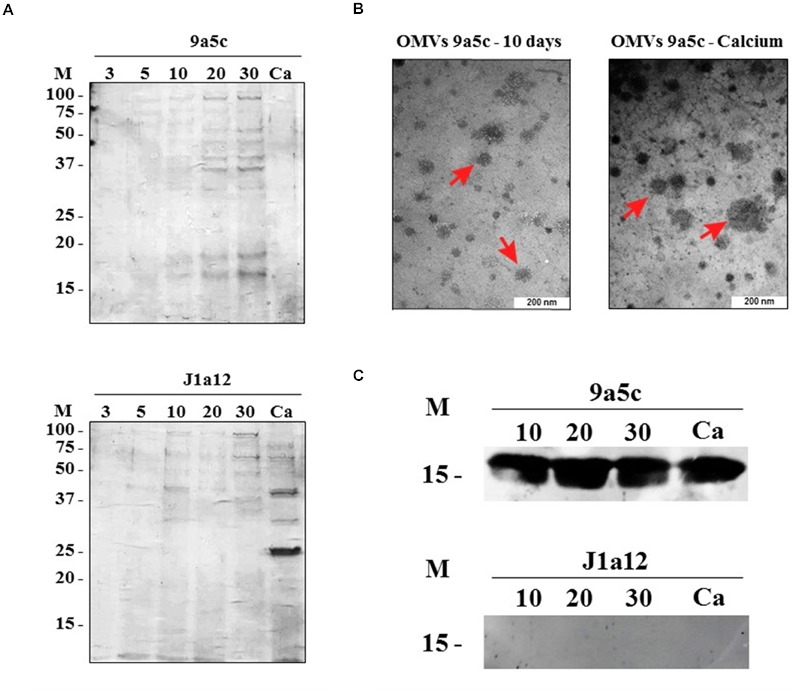
**(A)** SDS-PAGE of the total secreted proteins from *X. fastidiosa* strains 9a5c and J1a12. M: molecular weight marker (kDa); 3–30: days of growth; and Ca: 2.5 mmol dm^-3^ calcium (II) chloride. **(B)** Transmission electron microscopy of the outer membrane vesicles (OMVs) of *X. fastidiosa* strains 9a5c (red arrows indicate same of the OMVs in the photo). **(C)** Western blot analyses of the total protein inside the OMVs of *X. fastidiosa* strains 9a5c and J1a12. Proteins were identified using an antibody against Antitoxin (XF2491).

This work provides a detailed *in vitro* analysis of the extracellular proteins of *X. fastidiosa*. In this analysis, we used two strains, the virulent 9a5c strain, which is the causal agent of CVC in citrus trees in Brazil, and the J1a12 strain, which cannot form a thick biofilm and induce CVC symptoms when inoculated into citrus plants (Koide et al., 2004; Teixeira Ddo et al., 2004). *X. fastidiosa* was grown in PW medium (Davis et al., 1981), and the total protein content in culture broth, including the proteins associated with OMVs was visualized through long run SDS-PAGE (**Figure 2A**), while short run SDS-PAGE (migration only 1 cm into the gel) was used for mass spectrometry analysis (**Figure 1C**). The presence of OMVs was confirmed by ultracentrifugation and visualized by electron microscopy (**Figure 2B**).

**FIGURE 3:**
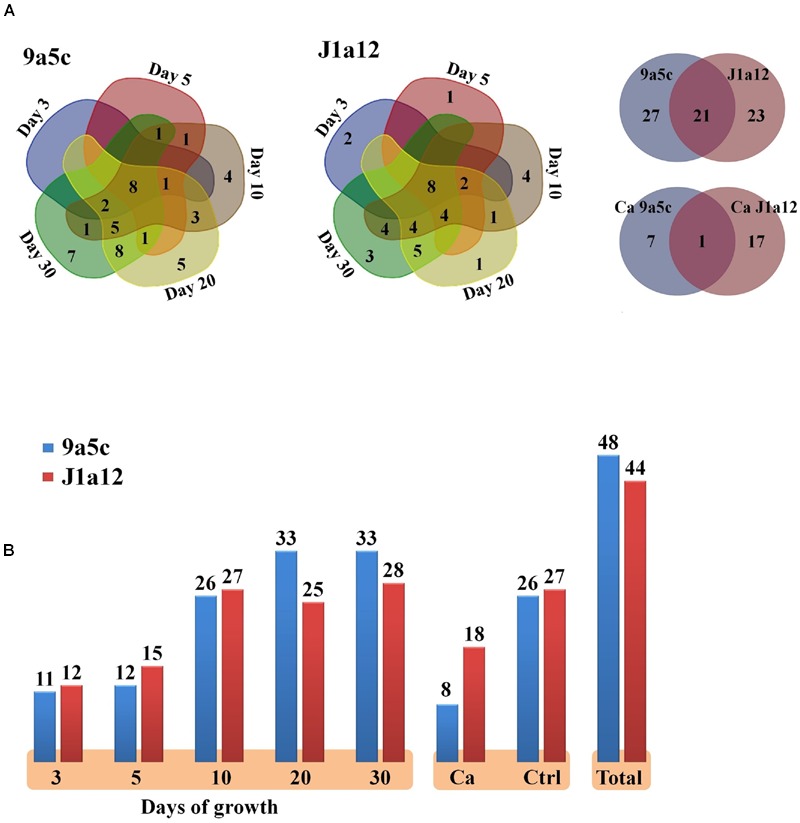
**Proteins secreted by strains 9a5c and J1a12 identified by MS. (A)** Distributions of the proteins on different days of growth (3–30 days) and distribution of the total proteins between the two strains. **(B)** Total proteins secreted on different days of growth, in response to 2.5 mmol dm^-3^ calcium (II) chloride (Ca) and in the respective control (Ctrl).

A previous studies of the *X. fastidiosa* proteome revealed differences between the planktonic and biofilm stages in the expression of proteins related to metabolism, motility, attachment, and stress conditions (Silva et al., 2011) and showed the presence of 30 extracellular proteins related to survival and pathogenesis after growth in solid medium for 21 days (Smolka et al., 2003; Silva et al., 2011). In the present study, we identified 48 proteins from strain 9a5c and 44 proteins from strain J1a12, which resulted in the identification of 71 non-redundant proteins, of which 21 were identified in both strains (**Figures 3A,B**). To identify the proteins in the text of the manuscript, we used the data bank and nomenclature of the *Xylella fastidiosa* Genome Project, performed by the Unicamp Laboratory for Bioinformatics (LBI-ID). However, in **Table 1**, we describe all of the data banks that could be used to search for each protein.

**Table 1:** Extracellular proteins of *Xylella fastidiosa.*

Protein	kDa	LBI-ID	LNCC-ID	UniProt^1^	SignalP^2^	TatP^3^	SecP^4^
60kDa chaperonin	57.8	XF0615	XF0494	Q9PFP2	No	No	0.077
Acetylornithine aminotransferase (ACOAT)	43.7	XF1427	XF1180	Q9PDF2	No	No	0.097
Adenylosuccinate lyase (ASL)	50.6	XF1553	XF1289	P44797	No	No	0.076
Amidohydrolase family protein	47.3	XF2472	XF2144	O66851	No	No	0.069
Aminopeptidase (peptidase M28 family protein)	57.7	XF0820	XF0671	O54697	No	No	0.529
Antitoxin (HTH-type transcriptional regulator)	15	XF2491	XF2163	Q46864	No	No	0.149
Autolytic lysozyme	23.4	XF2392	XF2065	P26836	No	No	0.432
Autotransporter beta-domain protein	79,2	XF2349	XF2033	-	Yes	No	0,875
Chaperone protein DnaK (heat shock protein 70)	68.5	XF2340	XF2024	Q9PB05	No	No	0.396
Conserved hypothetical protein	11.6	XF1971	XF1697	-	No	No	0.242
Conserved hypothetical protein	18.4	XF1941	XF1669	-	Yes	No	0.839
Conserved hypothetical protein	34.1	XF1434	XF1183	-	Yes	No	0.932
Conserved hypothetical protein	46.4	XF2151	XF1868	-	No	No	0.939
Conserved hypothetical protein	58.2	XF1384	XF1144	-	No	No	0.776
Conserved hypothetical protein	79.4	XF1887	XF1615	-	Yes	No	0.776
Conserved hypothetical protein, xfp6	37.6	XF0531	XF0426	-	No	No	0.897
Dihydrolipoyl dehydrogenase	50.7	XF1548	XF1285	P14218	No	No	0.065
Elongation factor Tu	42.9	XF2628	XF2288	Q9P9Q9	No	No	0.049
Endoribonuclease L-PSP family protein	13.6	XF0353	XF0275	P40431	No	No	0.694
Enolase (2-phosphoglycerate dehydratase)	45.8	XF1291	XF1064	Q9PDT8	No	No	0.07
Exoglucanase A (1,4-beta-cellobiohydrolase A)	70.9	XF1267	XF1049	P50401	No	No	0.836
Extracellular serine protease	105.4	XF1851	XF1585	P09489	No	Yes	0.952
Extracellular serine protease	95.2	XF1026	XF0844	P09489	No	No	0.868
Extracellular serine protease	95.8	XF0267	XF0216	P09489	No	No	0.948
FimX, fimbrial adhesin protein	19.2	XF0083	XF0063	P11312	Yes	No	0.95
Hypothetical protein	13	XF0898	XF0737	-	No	No	0.755
Hypothetical protein	13.7	XF2078	XF1798	-	No	No	0.943
Hypothetical protein	21.2	XF1803	XF1538	-	Yes	No	0.856
Hypothetical protein	32.4	XF0565	XF0455	P32793	Yes	No	0.494
Hypothetical protein	5.6	XF1631	-	-	No	No	0.052
Hypothetical protein	7	XF0899	-	-	No	No	0.961
Hypothetical protein	9.2	XF1217	XF1009	-	No	No	0.072
Hypothetical protein	9.9	XF2408	XF2080	-	No	No	0.056
Lipase/esterase	64.3	XF0781	XF0643	P40604	Yes	Yes	0.953
Metallo-beta-lactamase family protein	34.3	XF2283	XF1974	-	Yes	No	0.224
Organic hydroperoxide resistance protein	14.9	XF1827	XF1562	O68390	No	No	0.196
Outer membrane autotransporter Beta-domain protein	56.7	XF1264	XF1047	Q01443	No	No	0.939
Outer membrane lipoprotein Slp family	19.4	XF1811	XF1547	P76255	Yes	No	0.901
Outer membrane porin F (OmpA family protein)	40.1	XF0343	XF0272	P13794	Yes	No	0.937
Outer membrane protein FadL family	48.5	XF1053	XF0863	P80603	No	No	0.831
Outer membrane protein TolC	49.5	XF2586	XF2255	P02930	Yes	No	0.45
Outer membrane protein W (OmpW)	19.9	XF0872	XF0713	P21364	No	No	0.888
Pal protein (peptidoglycan-associated lipoprotein)	15.8	XF1896	XF1624	P07176	No	No	0.874
Peptidase S9 family protein	98.7	XF2551	XF2225	P39839	No	No	0.841
Peptidoglycan-associated outer membrane lipoprotein	15.7	XF1547	XF1284	P10325	No	No	0.826
Phage-related major capsid protein, xfp2	67.7	XF0714	XF2169	-	No	No	0.629
Phage-related protein	41.8	XF1704	XF1433	-	No	No	0.098
Phage-related protein, xfp3	36.5	XF1577	XF1314	-	No	No	0.538
Phage-related protein, xfp4	30.4	XF1649	XF1379	P76513	No	No	0.065
Phosphoserine aminotransferase (PSAT)	39.6	XF2326	XF2012	Q9PB19	No	No	0.163
PilA2 Tfp pilus assembly protein,	15.4	XF2539	XF2216	P17837	No	No	0.937
Porin O (POP)	43.7	XF0975	XF0803	P33976	No	No	0.717
POP O (POP)	45	XF0321	XF0260	P32977	Yes	No	0.487
Putative lipoprotein/OmpA family protein	25.8	XF0363	XF0282	P37665	Yes	Yes	0.365
Short chain dehydrogenase, giCVC	26	XF1726	XF1455	Q9LBG2	No	Yes	0.129
Succinyl-CoA synthetase, alpha subunit	29.6	XF2548	XF2222	P07459	No	No	0.647
Succinyl-CoA synthetase, beta subunit	41	XF2547	XF2221	Q9PAH1	No	No	0.1
Surface protein adhesin YadA-like XadA1	98.3	XF1516	XF1257	P05790	No	No	0.962
Surface protein adhesin YadA-like XadA-like protein)	118.5	XF1981	XF1707	P12021	No	No	0.947
TolB protein	47.6	XF1897	XF1625	Q9PC84	Yes	No	0.809
TonB-dependent receptor	103	XF2237	XF1940	P06129	Yes	No	0.929
TonB-dependent receptor	113.9	XF0550	XF0443	P06129	Yes	No	0.914
TonB-dependent receptor	96.3	XF2713	XF2357	P27772	Yes	No	0.947
TonB-dependent receptor	97.9	XF0339	XF0270	P06129	Yes	No	0.935
VirK protein	16.1	XF1945	XF1672	Q44433	Yes	No	0.898
Virulence protein (Virj family protein)	49	XF2679	XF2329	-	Yes	No	0.171
Xanthomonadales conserved hypothetical pal	51.7	XF0138	XF0106	Q9PH08	No	No	0.095
Xanthomonadales conserved hypothetical	17.9	XF0964	XF0796	-	No	No	0.359
Xanthomonadales conserved hypothetical	42.3	XF0357	XF0278	-	No	No	0.898
Xanthomonadales conserved hypothetical	42.6	XF0358	XF0279	-	No	No	0.898
Zinc metalloprotease (peptidase family M16 protein)	105	XF0816	XF0669	P55679	Yes	No	0.439

*LBI-ID: open reading frame from http://aeg.lbi.ic.unicamp.br/xf; LNCC-ID: open reading frame from http://www.xylella.lncc.br; ^1^uniprot accession number; ^2^secretion prediction according to Signal P; ^3^secretion prediction according to twin-arginine signal peptide cleavage sites in bacteria; ^4^secretion prediction according to SecretomeP. Numbers correspond to the signal peptide probability (score > 0.5 = positive prediction). The (–) symbol indicates no correspondence with sequences in the LBI-ID or LNCC-ID data banks. Proteins are listed in alphabetical order.*

The profile of the extracellular proteins of the 9a5c virulent strain revealed a distinct distribution during biofilm formation stages (**Figure 3B**). In the attachment stages corresponding to 3 and 5 days of growth, we identified 11 and 12 proteins, respectively, which were generally classified as outer membrane proteins, a serine protease and a fimbrial adhesin. On the tenth day, the number of extracellular proteins increased to 26, which indicated the presence of different enzymes and an increase in hypothetical proteins. After 20 days of growth (biofilm maturation stage), we identified 33 diverse proteins, which was the highest number of proteins identified among the different stages of biofilm formation. Similarly, after 30 days of growth (dispersion stage), 33 proteins were identified. In addition, eight proteins were observed at all stages of biofilm formation (XF1547, XF0343, XF0083, XF1851, XF0363, XF1577, XF0898, and XF1026), including a serine protease, the fimbrial adhesin FimX, Porin F and a peptidoglycan-associated outer membrane lipoprotein precursor. The largest number of exclusive proteins was observed on day 30 and then day 20 and day 10, and exclusive proteins were not detected on days 3 or 5 of growth.

The extracellular protein profile of the J1a12 strain also revealed a distinct distribution during the stages of development (**Figure 3B**). On days three and five of growth, we identified 12 and 15 proteins, respectively, which were classified as outer membrane proteins and fimbrial adhesins. On day 10 of growth, we identified 27 proteins, which included membrane proteins and many hypothetical proteins. However, the inability to form a thick and functional biofilm appeared to be correlated with the maintenance of protein levels in the subsequent stages of growth. On days 20 and 30, which corresponded to the mature biofilm and dispersion stages, 25 and 28 proteins were identified, respectively.

### Enzymes

**FIGURE 4:**
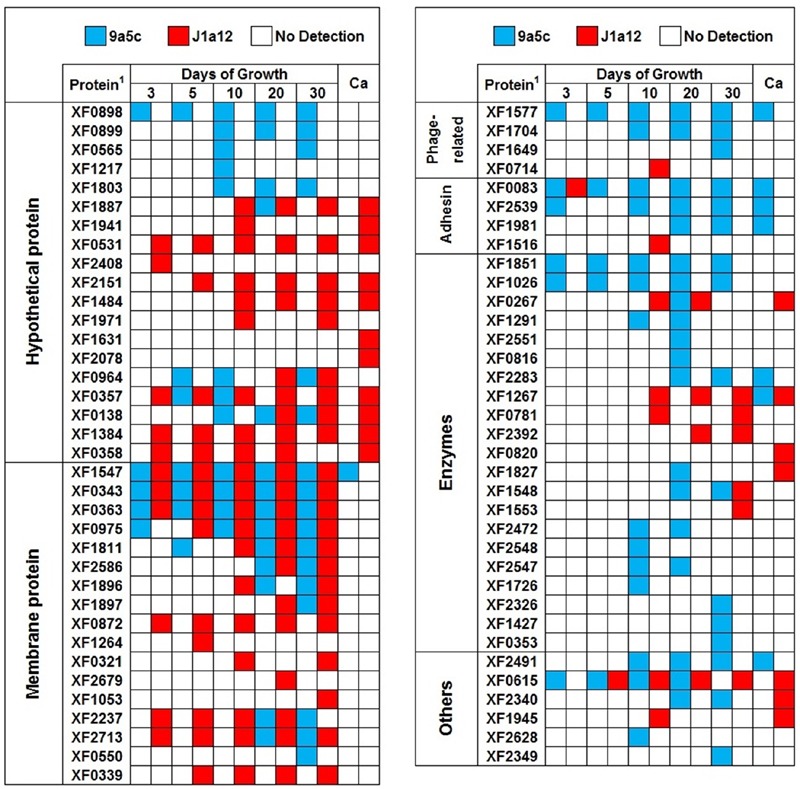
**Map of the secreted proteins identified on different days of biofilm growth and in response to Ca (2.5 mmol dm^-3^ of calcium (II) chloride) from *X. fastidiosa* strains 9a5c and J1a12.** ^1^The proteins are represented by their respective LBI-ID.

Enzymes are one of the most prevalent classes of secreted proteins in pathogenic bacteria, and their importance is principally related to their function in virulence. In this study, we identified a total of 21 enzymes with distinct functions, with 17 from strain 9a5c and eight from strain J1a12. Of these enzymes, 13 were exclusively found in strain 9a5c strain and four were exclusively found in strain J1a12 (**Figure 4**). Three serine proteases, XF1851, XF1026, and XF0267, were identified in 9a5c and one serine protease, XF0267, was identified in J1a12. The secreted proteases from this group were also identified in *in vitro* assays and found to be related to the dispersal of cells within the plant host (Fedatto et al., 2006). Furthermore, metallo-β-lactamase (XF2283) was only identified on day 20 and day 30 of growth in strain 9a5c and in response to calcium (II) treatment. Metallo-β-lactamase is widely distributed in Gram-negative bacteria and responsible for resistance to β-lactam antibiotics (Ambler, 1980; Wang et al., 1999; Heinz and Adolph, 2004). Metallo-β-lactamase is secreted into the bacterial periplasm or OMVs, thereby enabling extracellular β-lactam degradation (Pradel et al., 2009; Schaar et al., 2011; Devos et al., 2015).

### Other Protein Categories

**FIGURE 5:**
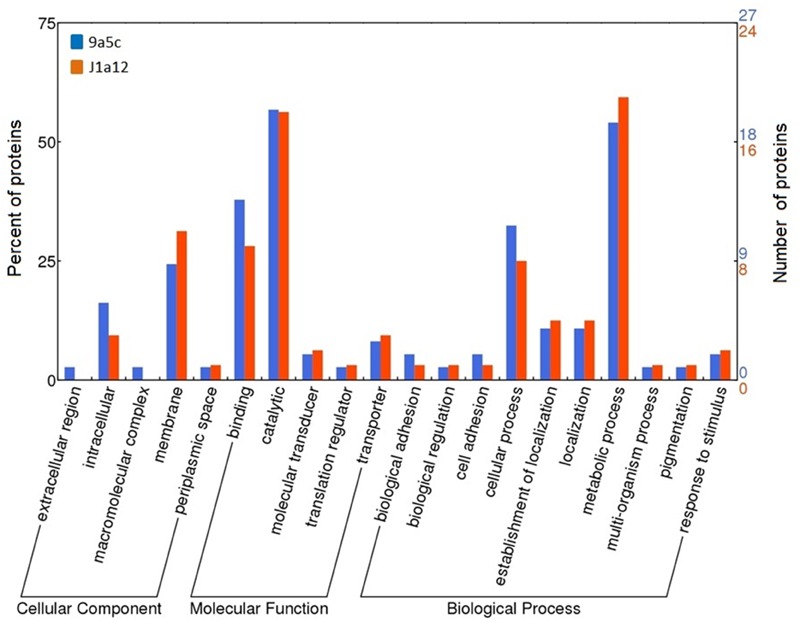
**Grouping of the identified secreted proteins according to the gene ontology terms cellular component, molecular function and biological process**.

All of the identified proteins are compiled in **Figure 4**, which indicates the stage of biofilm development (days) at which they were identified and whether they were observed during calcium (II) treatment. The results of the protein annotation and prediction analyses are presented in **Table 1**. Within the combined extracellular proteins of 9a5c and J1a12, we identified 19 proteins that were previously classified as hypothetical, with five found exclusively in 9a5c, 10 found exclusively in J1a12, and four found in both strains. These results emphasize that the function of these new proteins must be characterized based on their importance in the pathosystem. The identified proteins with known functions were grouped according to the gene ontology terms cellular component, molecular function and biological process (**Figure 5**).

We identified a group of membrane proteins that have been reported to be related to the structure of the OMVs, and they included the OmpF, OmpA, and OmpW proteins (Sidhu et al., 2008; Deatherage et al., 2009); TolB and Pal proteins (Bernadac et al., 1998); peptidoglycan-associated outer membrane lipoprotein (Wessel et al., 2013); the FadL family proteins (Bauman and Kuehn, 2006); and TonB-dependent receptor protein (Veith et al., 2014). However, more specific studies are necessary to relate these proteins to the formation of OMVs in *X. fastidiosa*. OMVs are spherical membrane bilayers composed of lipids, proteins, lipopolysaccharides and other molecules derived from the outer membrane of the bacterium; thus, they may incorporate many of surface elements (Demuth et al., 2003; Parker and Keenan, 2012; Kulkarni and Jagannadham, 2014). Different molecules inside OMVs are secreted and have distinct functions, including bacterial defense functions against antibacterial molecules and bacteriophage attacks, and a number of these molecules might induce OMVs and remove the phage before its DNA is injected (Kulp and Kuehn, 2010; Manning and Kuehn, 2011), promoting virulence, and pathogenicity (Jang et al., 2014). Additionally, four phage-related proteins were identified (XF1577, XF1704, XF1649 only in 9a5c; and XF0714 only in J1a12).

Adhesins, such as fimbrial FimX (XF0083) and PilA2 (XF2539), which are components of type IV pilus biogenesis, and non-fimbrial XadA (XF1981) were identified in the 9a5c strain, while XadA1 (XF1516) was identified in J1a12. Adhesins are frequently identified in bacterial secretome studies (Smolka et al., 2003; Kaakoush et al., 2010; Indrelid et al., 2014). However, studies in *X. fastidiosa* have shown that low concentrations of BSA present in the PW medium during *in vitro* growth reduce biofilm formation and stimulate the development of the widest fringe (Galvani et al., 2007). As BSA was not used in our methodology, it is important to consider the presence of PilA (XF2539), identified only in the 9a5c strain, as possible a result of this correlation. Although adhesins are often found at the surface of the cell membranes, XadA1 has been observed in the OMVs of the *X. fastidiosa* Temecula strain (Ionescu et al., 2014).

### Calcium Supplementation

Calcium supplementation changed the pattern of identified proteins in the extracellular medium of *X. fastidiosa*. Eight proteins were identified, including four adhesins (XF0083, XF2539, XF1981, and XF1516) and one hydrolase (exoglucanase A, XF1267) (**Figures 3** and **4**). Interestingly, this hydrolase was not observed during the development stages of biofilm formation without calcium. Overall, under normal conditions without calcium supplementation (control), 26 proteins were identified, including serine proteases (XF1851 and XF1026), an amidohydrolase (XF2472) and an enolase (XF1291). The decrease in extracellular proteins in the 9a5c strain was also apparent in the SDS-PAGE analysis of total extracellular proteins (**Figure 2A**). In both analyses, differences were observed between the treatments. The J1a12 strain also exhibited a decrease in extracellular proteins compared with the control, with the number decreasing from 27 to 18 when cultured in medium supplemented with calcium (II).

### Antitoxin Extraction and Identification Inside Outer Membrane Vesicles

Recombinant antitoxin (XF2491) was successfully expressed and purified through Ni-NTA affinity chromatography for the synthesis of a specific anti-rabbit IgG polyclonal antibody by Rhea Biotech, Campinas, Brazil. After OMV isolation via ultracentrifugation and confirmation of their existence using transmission electron microscopy (**Figure 2B**), the total inner content of the OMVs was extracted for analysis by Western blotting. The results demonstrated the presence of the antitoxin in the 9a5c strain from 10 to 30 days of growth and under calcium treatment; however, the J1a12 strain did not exhibit this protein within the analyzed OMVs (**Figure 2C**). Although some residual cell lysis may have occurred, this result represents an important validation method for verifying the presence of proteins from any secretion system in the extracellular medium under intense biofilm formation, disregarding a total false positive through contamination and making new approaches for studying the pathogenicity of *X. fastidiosa* possible.

## Discussion

It has previously been documented that the different growth stages of *X. fastidiosa in vitro* (planktonic to biofilm formation to cell dispersion) occur in a cycle with a duration of approximately 30 days (de Souza et al., 2004). According to the expected profile, studies of *X. fastidiosa* subsp. pauca strain 9a5c have used the specific days of growth to analyze the expression profiles of genes and proteins in the planktonic stage and biofilm formation stage, and the results have revealed significant differences between the analyzed stages (de Souza et al., 2004; Caserta et al., 2010; Toledo et al., 2013; Mendes et al., 2015). Similarly, *X. fastidiosa* strain J1a12 has been used for comparison with the virulent strain (9a5c). J1a12 cannot form a thick biofilm or induce CVC symptoms when inoculated into citrus plants, despite exhibiting a 94.5% conserved coding sequence compared with the 9a5c strain (Koide et al., 2004; Teixeira Ddo et al., 2004). Thus, the same parameters were applied in our work to resolve the mechanisms underlying biofilm formation of *X. fastidiosa* over a period characterized by changes in the stage of the cell growth as described in previous studies. Therefore, *X. fastidiosa* was analyzed on five different days of growth (3, 5, 10, 20, and 30) in the present study (**Figure 2A**). Each day corresponds to a different stage of *X. fastidiosa* development on an abiotic surface (glass Erlenmeyer flask), as follows: reversible attachment (3), irreversible attachment (5), initial biofilm maturation (10), total maturation (20), and cell dispersion (30) (de Souza et al., 2004; Caserta et al., 2010).

By analyzing the results regarding the distribution of proteins by categories and strains (**Figure 4**), it was possible to identify what was shared and what differed between 9a5c and J1a12 in this analysis. In the *Hypothetical Protein* section, there were two categories, one including a group of exclusive proteins identified in either 9a5c or J1a12, while the other group of proteins are found in both strains. Based on these results, an important field of research can be initiated, with the aim of identifying new possible virulence factors involved in *X. fastidiosa* pathogenicity. In the *Membrane Protein* section, there was a predominance of proteins found in both strains and proteins exclusive to the J1a12 strain. We believe that these membrane proteins are related to the structure of OMVs, which are derived from the outer membrane of the bacterium. This hypothesis can be confirmed by the results of a previous study on the proteome of the OMVs of *Xanthomonas campestris*, in which the same membrane proteins identified in our study were found, such as OmpA, OmpW, FadL, and the TonB-dependent receptor (Sidhu et al., 2008). In the *Phage-related* and *Adhesin* sections, the 9a5c strain showed greater prominence in all stages of growth. The greater representation of adhesins is probably due to the lower production of biofilm of J1a12, making it incapable of inducing CVC symptoms when inoculated onto citrus plants; on the other hand, the presence of toxins and virulence-associated proteins within prophage-like elements in the *X. fastidiosa* genome (de Mello Varani et al., 2008) can explain the increased presence of phage-related proteins in the 9a5c strain. Similarly, in the *Enzymes* section, the presence of these proteins was highest in the 9a5c strain, probably due to their greater role in pathogenicity. Studies have shown that LesA secretion is an important virulence factor in *X. fastidiosa*, the causal agent of Pierce’s disease in grapevines (Nascimento et al., 2016). We hypothesize that LesA (XF0781) is also one of the most important virulence factors in *X. fastidiosa* 9a5c, in addition to three identified extracellular serine proteases, acting in the degradation of pit membranes and allowing spreading within xylem vessels through bordered pits. We identified some cytoplasmic proteins, such as elongation factor Tu (XF2628), a 60 kDa chaperonin (XF0615), and DnaK (XF2340). These proteins were identified in previous studies examining the proteome of the OMVs of *Brucella melitensis* (Avila-Calderón et al., 2012), *Yersinia pestis* (Eddy et al., 2014), and *Edwardsiella tarda* (Park et al., 2011). Elongation factor Tu is secreted in the OMVs of the Gram-negative bacterium *Burkholderia pseudomallei* and has been suggested to show chaperone properties in *E. coli* (Caldas et al., 1998; Nieves et al., 2010). However, more specific assays must be performed to identify the function of each of these proteins in *X. fastidiosa*.

Based on the results of 1D SDS-PAGE (**Figure 2A**), we observed an increase in visible bands in the course of biofilm growth in both strains. This result was representative of the quantitative results obtained through mass spectrometry (**Figure 3B**), Similarly, calcium treatment resulted in a decrease in visible bands in 9a5c. In J1a12 under calcium treatment, a smear could be observed from the protein band marker below 25 kDa, and two bands of approximated 37 and 25 kDa could be highlighted. However, overlapping distributions between bands should be considered.

Our protein secretion predictions guided us to investigate the secretion system of *X. fastidiosa in vitro*. We used three secretion prediction tools. *SignalP* (Nielsen et al., 1997) predicts the classical signal peptide cleavage sites, *SecretomeP* (Bendtsen et al., 2005a) is based on the non-classical secretory pathway and predicts proteins without an N-terminal signal peptide, and *TatP* (Bendtsen et al., 2005b) predicts twin-arginine signal peptide cleavage sites. In all of the analyses, 53 proteins had a secretory function in at least one prediction test. However, 21 proteins did not have a positive prediction in at least one of the tests performed, among which six were found to be *X. fastidiosa* hypothetical proteins. Thus, contamination from residual cell lysis should be considered. However, OMVs are often used as a vehicle to transport proteins during the growth of *X. fastidiosa*, which led us to consider that the identified proteins were delivered to extracellular space through secretion mechanisms. To corroborate this hypothesis, the presence of antitoxin (XF2491) was evaluated. After isolation of the OMVs (**Figure 2B**) and extraction of the proteins from vesicles, Western blot analysis using an antibody against this protein confirmed that antitoxin (XF2491) was secreted into the culture medium via the OMVs from day 10 until day 30, which is equivalent to the dispersion stage, and during calcium (II) treatment. On the other hand, antitoxin was not identified inside the OMVs of strain J1a12 (**Figure 2C**). This antitoxin is an HTH-type transcriptional regulator and is a member of a type II toxin-antitoxin system; it resides in the same operon as the XF2490 toxin. *X. fastidiosa* strain Temecula homologues correspond to MqsR/YgiT and function to regulate population levels (Lee et al., 2014). Among other functions, the toxin-antitoxin system is related to persistent cell production, biofilm formation, and stress responses in *X. fastidiosa* (Muranaka et al., 2012) as well as in other bacteria (Wang and Wood, 2011; Merfa et al., 2016). Moreover, we believe that certain proteins may utilize a similar secretion system to the antitoxin (XF2491) within OMVs.

*Xylella fastidiosa* uses the quorum-sensing signal DSF, which is associated with cellular density, during the expression of pathogenicity-related genes (Scarpari et al., 2003; Beaulieu et al., 2013). In this study, this signal was indispensable for the analysis of the different stages of growth and provided a broad base of qualitative and quantitative data that correspond to the entire process of *X. fastidiosa* biofilm development. These data were compared with data obtained in previous studies that investigated subsp. pauca strain 9a5c. In studies on *X. fastidiosa*, the slow growth of this fastidious bacterium must be considered. *In vitro*, the biofilm aggregates are located in the middle of the flask walls and primarily formed from dead cells (Chatterjee et al., 2008). Thus, to prevent cellular debris from contaminating the suspension, the media were centrifuged at 16,000 rpm and filtered through a 0.22 μm membrane before SDS-PAGE electrophoresis was performed to avoid false positives in the posterior mass spectrometry analyses. Despite these precautions, residual cell lysis should be considered.

Previous studies have demonstrated that calcium increases the surface attachment, biofilm formation, and twitching motility of *X. fastidiosa* strain Temecula under *in vitro* conditions (Cruz et al., 2012, 2014). Similarly, Parker et al. (2016) showed that calcium supplementation changed the global expression of genes that promote continued biofilm development. These genes are related to attachment, motility, exopolysaccharide synthesis, biofilm formation, peptidoglycan synthesis, regulatory functions, iron homeostasis, and phages. Although biofilm formation is an important aspect of the pathogenicity of *X. fastidiosa*, particularly the appearance of symptoms of xylem vessel occlusion, studies have shown that the greatest degree of virulence is dependent on the movement of cells within the host (planktonic cells) (Guilhabert and Kirkpatrick, 2005). Corroborating our results, the increase in biofilm formation induced by calcium supplementation appears to block the secretion of proteins, thereby contributing to a drastic reduction of virulence. This behavior was also observed in *Y. pestis* grown *in vitro* in the presence of external calcium (Fowler and Brubaker, 1994; Fowler et al., 2009). The presence of calcium and the virulence of *X. fastidiosa* infection appear to be closely correlated. *In vivo* studies have shown that the calcium balance changes in plant hosts infected by *X. fastidiosa*. An increase in the calcium concentration was detected in *Nicotiana tabacum* leaves prior to the appearance of symptoms (De La Fuente et al., 2013). Histological studies of *C. sinensis* and *Coffea arabica* infected with *X. fastidiosa* revealed an accumulation of calcium oxalate crystals in occluded vessels (Queiroz-Voltan et al., 1998; Alves et al., 2009). Calcium oxalate crystals are insoluble structures formed from the free calcium (II) and endogenous oxalic acids that are present in many plants. These structures have a number of functions, including the regulation of excess calcium (Franceschi and Nakata, 2005).

Finally, the methodology employed in this study considered the intrinsic behavior of *X. fastidiosa*. The slow growth of the bacterium was a great challenge when normalizing all of the biofilm growth processes. In this study, we did not intend to analyze the protein expression levels because this is a preliminary study that was designed to evaluate the overall extracellular protein profile of two different strains of *X. fastidiosa*. Thus, we believe that our results add important information on the molecular mechanisms used by *X. fastidiosa*, especially in the secretion system under abiotic conditions, and contributes to our understanding of the molecular mechanisms responsible for the behavior of this pathogen in the host.

## Conclusion

Virulence and pathogenicity depend on a plethora of bacteria–host interactions and involve different gene products. In the present study, we performed a descriptive analysis of the extracellular proteins identified in two strains of *X. fastidiosa* (9a5c and J1a12) grown on PW culture media at different stages of biofilm formation. This assessment of the behavior of *X. fastidiosa* provided insights that can be used to advance our understanding of the host–pathogen interactions. The presence of a strain that cannot form a thick biofilm and induce CVC symptoms (J1a12) allowed us to compare and highlight extracellular proteins of the virulent strain (9a5c) correlated with robust biofilm formation.

Our results highlight the presence of different enzymes related to cell wall degradation and membrane proteins, all of which may be used in biofilm growth and maintenance and dispersal within the xylem vessel. Moreover, the large number of identified proteins that were previously classified as hypothetical demonstrates that a functional study of these proteins must be performed to better understand the mechanism of *X. fastidiosa* pathogenicity. In addition, we demonstrated that supplementation with calcium induces a drastic reduction of protein secretion. The protein secretion predictions used in this work allowed us to better evaluate the potentially secreted proteins that are usually identified in the *X. fastidiosa* secretion system via mass spectrometry. However, the presence of the antitoxin (XF2491) secreted by OMVs and the absence of a positive prediction in at least one of the tests performed showed us that there are a wide range of responses related to the pathogenicity of *X. fastidiosa* to be elucidated. Although *in vivo* studies of the secretome of the interactions between *X. fastidiosa* and its host plant are required, we believe that this work provides insights into these interactions that will support new approaches in *X. fastidiosa* research.

## Author Contributions

JM, AS, MH, MT, AAdS, LT, and APdS contributed to the conception and design of the work; JM and AS performed the data acquisition and designed experiments; JM, AS, MH, MT, AAdS, LT, and APdS drafted the article or revised it critically for important intellectual content. All authors read and approved the final manuscript.

## Conflict of Interest Statement

The authors declare that the research was conducted in the absence of any commercial or financial relationships that could be construed as a potential conflict of interest.

## Footnotes

1 http://aeg.lbi.ic.unicamp.br/xf/

2 http://www.xylella.lncc.br/xf-webbie/final/main.html#
